# A *fast and frugal* algorithm to strengthen diagnosis and treatment decisions for catheter-associated bacteriuria

**DOI:** 10.1371/journal.pone.0174415

**Published:** 2017-03-28

**Authors:** Aanand D. Naik, Felicia Skelton, Amber B. Amspoker, Russell A. Glasgow, Barbara W. Trautner

**Affiliations:** 1 Houston Center of Innovations in Quality, Effectiveness, and Safety, Michael E DeBakey Veterans Affairs Medical Center, Houston, Texas, United States of America; 2 Health Services Research Section, Department of Medicine, Baylor College of Medicine, Houston, Texas, United States of America; 3 Department of Family Medicine, University of Colorado Anschutz Medical Campus, Denver, Colorado, United States of America; Azienda Ospedaliera Universitaria di Perugia, ITALY

## Abstract

**Objectives:**

Guidelines for managing catheter-associated urinary tract infection (CAUTI) and asymptomatic bacteria (ASB) are poorly translated into routine care due in part to cognitive diagnostic errors. This study determines if the accuracy for CAUTI and ASB diagnosis and treatment improves after implementation of a fast and frugal algorithm compared with traditional education methods.

**Materials and methods:**

A pre and post-intervention with contemporaneous comparison site involving inpatient and long term care wards at two regional Veterans Affairs Systems in United States. Participants included 216 internal medicine residents and 16 primary care clinicians. Intervention clinicians received training with a fast and frugal algorithm. Comparison site clinicians received guidelines education. Diagnosis and treatment accuracy compared with a criterion standard was assessed during similar three-month, pre- and post-intervention periods. Sensitivity, specificity, and likelihood ratios were compared for both periods at each site.

**Results:**

Bacteriuria management was evaluated against criterion standard in 196 cases pre-implementation and 117 cases post-implementation. Accuracy of bacteriuria management among intervention participants was significantly higher, post-implementation, than those at the comparison site (Intervention: positive likelihood ratio (LR+) = 8.5, specificity = 0.89, 95% confidence interval (CI) = 0.78−1.00; comparison: LR+ = 4.62, specificity (95%CI) = 0.79 (0.63−0.95). Further, improvements at the intervention site were statistically significant (pre-implementation: LR+ = 2.1, specificity (95%CI) = 0.60 (0.50−0.71); post-implementation: LR+ = 8.5, specificity (95%CI) = 0.89 (0.78−1.00). At both sites, there were similar improvements in negative LR from pre- to post-implementation: [Intervention site = 0.28 to 0.08; comparison site = 0.13 to 0.04]. Inappropriate management of ASB declined markedly from 32 (40%) to 3 (11%) cases at the intervention site.

**Conclusions:**

A fast and frugal algorithm improves diagnosis and treatment accuracy for CAUTI and reduces inappropriate treatment of ASB. Fast and frugal algorithms that realign diagnostic intuitions and treatment norms can enhance translation of evidence into practice.

## Introduction

Bacteriuria in patients with urinary catheters presents as symptomatic catheter-associated urinary tract infection (CAUTI) or asymptomatic bacteriuria (ASB). Failure to distinguish between these two conditions results in a frequently observed gap between evidence-based guidelines and routine care [1]. Catheter-associated bacteriuria is common in hospital settings and more often presents as ASB [2]. Treatment for ASB in most scenarios is unlikely to confer benefits and may be potentially harmful, contributing to resistant organisms, adverse drug events, and *Clostridium Difficile* infection [2–4]. The Infectious Diseases Society of America (IDSA) guidelines recommend clinicians neither screen for nor treat ASB in most catheterized patients; these guidelines were also endorsed by the United States Preventative Services Task Force (USPSTF) and the Choosing Wisely Campaign [3, 5–7]. Despite these recommendations, studies document 20% to 83% of patients with ASB are treated unnecessarily with antibiotics [2, 8]. De-implementation of wasteful, ineffective or harmful practices often requires specific commitment to evidence-based practice [9]. Antibiotic overuse runs counter to national efforts to stem the emergence of highly resistant bacteria through antibiotic stewardship [10]. However, translating ASB and CAUTI guidelines appears difficult for many when faced with “positive” urinalysis or urine culture results [11–12].

### Why is it hard to adopt guidelines into practice?

Clinical practice guidelines (CPG) are the preferred method among professional societies for defining evidence-based, high quality health care [13]. Despite widespread development and dissemination of CPG by professional societies and independent bodies like the United States Preventive Services Task Force (USPSTF), their impact on routine practice remains suboptimal [14]. The complexity of CPGs (i.e., ASB and CAUTI guidelines are 51 pages in length) or their incompatibility with pre-existing treatment norms and diagnostic intuitions, may limit their uptake [15]. As a result, experts typically recommend multi-component implementation systems for CPG [11–12, 16]. While effective, these overly-complex strategies themselves become barriers to rapid adoption in routine care [15].

Our hypothesis is that CPG can be implemented by applying two elegant simplifications. The first focuses on narrowing the teachable aspects of CPG to the fewest set of decision cues essential for changing practice (guideline adoption). Experimental studies demonstrate that expert clinicians rely more on intuitive judgments based on recognition of familiar patterns of variables within an environmental context (i.e., clinical cues) that allow for rapid and largely accurate decisions without having to explicitly compare options [17–18]. In essence, the design of most CPGs is too clumsy and cumbersome for the *fast and frugal* nature of medical decisions [19–20]. The second simplification arises from an awareness of how CPGs are used contextually at the point of care (guideline implementation). If the decision-making steps are kept simple and few in number, then the intervention necessary for facilitating implementation at the point of care may likewise avoid complexity.

### The Evidence Integration Triangle

**Fig 1:**
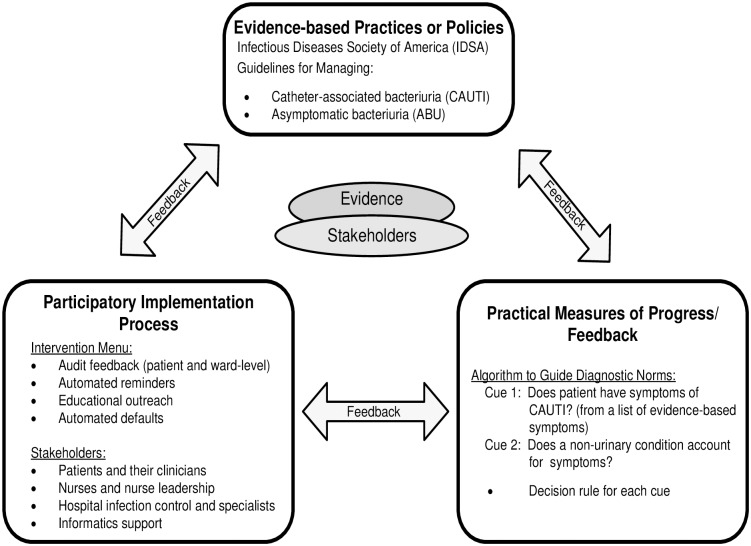
Evidence Integration Triangle (EIT) for rapid adoption of clinical practice guidelines. Evidence Integration Triangle adapted for rapid adoption of the Infectious Diseases Society of America clinical practice guidelines for catheter-associated urinary tract infection and asymptomatic bacteriuria into routine care. This adaptation is modified from the original EIT model.

We suggest the Evidence Integration Triangle (EIT) [21] as a framework to guide these two simplifications for evidence adoption and implementation (Fig 1). We have previously developed the Kicking-CAUTI campaign [22] using the EIT framework to enhance management of CAUTI and ASB at the point of care in inpatient and long-term care settings. For the Kicking-CAUTI intervention, the CAUTI and ASB clinical practice guidelines serve as the top point in the EIT framework. The lower two points describe how these guidelines can be adopted by clinicians (right corner) and implemented (left corner) into care.

The right corner of the EIT (Fig 1) includes “practical measures” for guiding CPG adoption. In the EIT model, practical measures are most effective when they are intuitive and promote action [21]. We suspect that one of the main reasons that ASB is so often treated inappropriately with antibiotics is that ASB guidelines are not consistent with clinicians’ existing norms [19] and intuitive judgements [20] for diagnosis and treatment. Withholding antibiotics goes against clinical norms when laboratory results show the patient’s urine has a high white blood cell count (pyuria) or is growing an organism with pathogenic potential (gram-negative rods) [19, 23]. To facilitate de-implementation for Kicking-CAUTI, we developed a fast and frugal algorithm to realign diagnostic intuitions for catheter-associated bacteriuria with CPGs [20, 23]. Based on the theory of fast and frugal heuristics, these algorithms rely on the fewest number of necessary decision cues and present choices in ways that permit rapid processing [20, 23]. Our algorithm distills the process for distinguishing between CAUTI versus ABU down to two decision cues with simple rules for each cue (Fig 1). An intervention using the algorithm as its decision support tool may realign diagnostic intuitions towards CPGs while correcting treatment errors.

The left corner of the EIT consists of a “participatory implementation process” (Fig 1). This process clarifies the “who, when, where, how, and why” of guidelines using the range of interventions described in Fig 1. For Kicking-CAUTI, inpatient and long-term care clinicians received personalized audit and feedback at their clinical settings that compared their urine culture ordering and treatment actions to the guidelines standards using the fast and frugal algorithm as a training reference [22]. To close the EIT triangle, proposed future revisions of the ASB guidelines will include stakeholder suggestions from the Kicking-CAUTI study [24].

We previously reported on the implementation processes and outcomes of Kicking-CAUTI as demonstrated by lower rates of urine culture ordering versus a comparison site (Incidence rate ratio = 0.57 versus 0.29; p <.001) and overtreatment of ASB using personalized audit and feedback [25]. We have not previously reported on how successful the algorithm was at realigning diagnostic intuitions and correcting treatment norms for managing CAUTI and ASB (right corner of EIT). This study evaluates the predictive validity of a fast and frugal algorithm to improve diagnosis and treatment of catheter-associated bacteriuria following algorithm adoption compared to baseline and compared with changes at the comparison site.

## Materials and methods

### Participants

This study was reviewed and approved by the Baylor College of Medicine Institutional Review Board and the University of Texas Health Science Center at San Antonio Institutional Review Board. The protocol and procedures were also approved by Research and Development Committees of the Michael E. DeBakey Veterans Administration Medical Center and the Audie Murphy Memorial Veterans Health Care System. Waiver of consent was approved by institutional review boards, and the study did not include minors.

The participants of the current study were drawn from the health care providers who participated in the Kicking-CAUTI project, previously described in greater depth [22, 26]. The participants of the current study were healthcare providers on general acute medicine and long-term care wards in two Veterans Affairs Hospitals during two three-month time periods before (Nov 2010-Jan 2011) and after intervention implementation (Nov 2011-Jan 2012). The intervention was initiated in July 2011, therefore our evaluation period allows for a few months of intervention uptake along with a calendar-matched baseline period. The intervention targeted all healthcare providers who make decisions to screen for or treat CAUTI. In the hospital medicine wards, this decision is made by physician teams, usually composed of either categorical medicine residents, medicine-pediatrics residents, or preliminary year medicine residents doing a medicine rotation (including anesthesiology, psychiatry, neurology, and transitional year residents). In the long term care wards, the intervention targeted teams of staff nurses, nurse practitioners, physician assistants, and physicians.

### Fast and frugal algorithm

**Fig 2:**
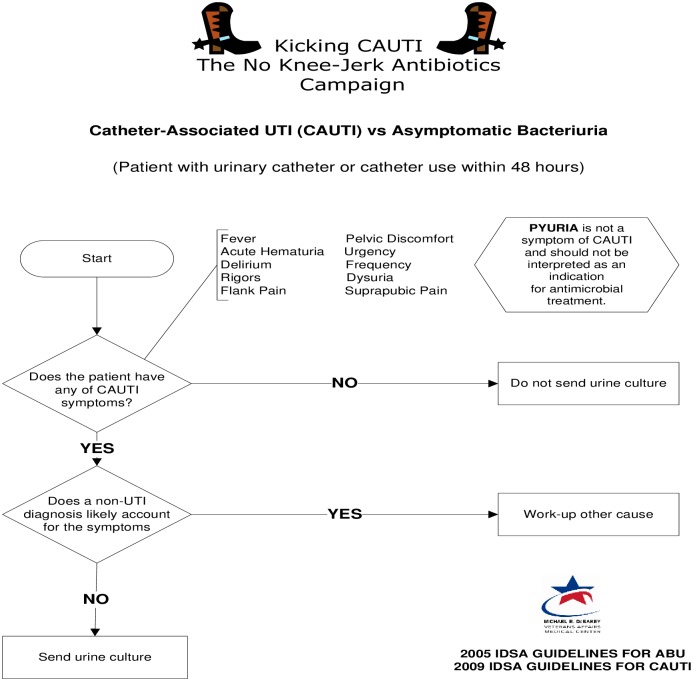
Fast and frugal diagnostic algorithm for differentiating Asymptomatic Bacteriuria (ABU) versus Catheter Associated Urinary Tract Infections (CAUTI). Fast and Frugal algorithms follow these three simple rules: 1) Search Rule: Search through cues in a predetermined order. Cue 1: Are there evidence-based symptoms of CAUTI present? Cue 2: Is there a non-urinary cause for these symptoms? 2) Stop Rule: Stop after the first and second cues to discriminate between alternatives (ABU versus CAUTI). 3) Decision Rule (classify the episode accordingly): If the answer to cue 1 is negative then ABU is more likely. If cue 1 is positive but cue 2 is negative, then CAUTI is more likely. The Kicking CAUTI algorithm also contains an explicit corrective for cue 1 to counteract the most common cognitive bias in distinguishing between ABU and CAUTI: "Pyuria is not a symptom of CAUTI and should not be interpreted as an indication for antimicrobial treatment."

In conditions of uncertainty that require rapid decision making, experts often rely on heuristics (mental short-cuts and rules of thumb) to make fast, frugal, and effective decisions. Heuristics help experts to examine fewer cues from the environment, simplify the process of weighing and valuing cues, and reduce time and effort to examine alternative options and make choices [20]. Because of these simplifications, heuristics are also prone to bias and error, even among experts [18]. Expert decision making can be augmented with the use of decision support tools such as *fast and frugal* algorithms [20]. In contrast to other decision algorithms that are typically optimizing in nature (processing all variables), fast and frugal algorithms are based on the concept of satisficing (simplifying to essential variables)—a theoretical guide for “less is more” in healthcare. Such algorithms support expert decision making by specifying three decision making rules: (1) search rule to specify in what direction a search for cues extends, (2) stopping rule to specify when the search is stopped, and (3) decision rule to specify how the final decision is reached [20]. The current analysis focuses specifically on the validation of a *fast and frugal* algorithm (see Fig 2 with search, stop, and decide rules) as an index test for improving the management of CAUTI and ASB (diagnosis and treatment). This algorithm also includes specific correctives for common sources of bias including the guidelines-discordant cue of pyuria.

### Study design and test methods

The study design consisted of a pre- and post-intervention comparison with a contemporaneous control. The intervention group received the algorithm (Fig 2) and case-based training on its use. We have previously described the development, refinement and preliminary validation of the algorithm [23] and the training process [22,25]. All hospital physicians and long-term care providers enrolled at the Kicking-CAUTI intervention site were given copies of the algorithm and case-based training on how to apply it to patients with catheter associated bacteria. The case based training was conducted by research staff and typically lasted 15–20 minutes. Training targeted teams of clinicians in long-term care or acute inpatient medical care and focused on walking clinicians through each of the decision rules within the algorithm as it applied to specific cases in which urine cultures were ordered. The comparison group received standard education about CAUTI guidelines, but not the fast and frugal algorithm. The clinicians who made the decision to initiate antibiotics were often the clinicians who ordered the urine culture, but in some cases the urine culture was ordered by the emergency room providers.

#### Positive urine culture in patients with urinary catheters

At both the intervention and comparison sites, study personnel evaluated all catheter associated urine culture orders among patients with urinary catheters in place >48 hours during the targeted pre and post implementation periods. For each positive urine culture (defined as ≥10^3^ organisms/mL of urine) reported by the microbiology lab from one of the study wards, study personnel first identified whether a urinary catheter was present, and, if so, classified the case as either CAUTI or ASB.

#### Reference standards

CAUTI is defined as the presence of at least 1 of the following signs and symptoms with no other recognized cause: fever (≥100°F), urgency, frequency, dysuria, suprapubic tenderness, pelvic discomfort, costovertebral angle tenderness, hematuria, rigors, or delirium in a patient with a positive urine culture who had a urinary catheter within the past 48 hours [24, 26]. In contrast, ASB is defined as a positive urine culture without signs and symptoms consistent with the definition of CAUTI [25,27]. The criterion standard for appropriate management for CAUTI versus ASB is based on expert assessment of the diagnosis and treatment of each type of bacteriuria [25,27]. For confirmed cases of CAUTI, treatment with antibiotics was appropriate, while failure to use antibiotics was inappropriate. For confirmed cases of ASB, the use of antibiotics was inappropriate, and withholding antibiotics was appropriate.

### Data analysis

Baseline characteristics of the study participants were reported using descriptive statistics. We assessed CAUTI and ASB management accuracy of participants using measures of sensitivity, specificity and likelihood ratios. Using the standard 2x2 table for diagnostic statistics [28], all cases were fit into one of four categories: true positive, false positive, true negative, and false negative. True positives are defined as episodes of positive urine cultures in a symptomatic patient that were treated with antibiotics. True negatives are defined as episodes of positive urine cultures in asymptomatic patients that were not treated with antibiotics. False positives are defined as episodes of positive cultures in asymptomatic patients but treated with antibiotics. False negatives are defined as episodes of positive cultures in symptomatic patients for which no antimicrobials were prescribed.

Sensitivity was calculated as those with symptoms who received treatment (true positives) divided by treated and untreated patients with symptoms (true plus false positives). Specificity was calculated as those not treated and without symptoms (true negative) divided by treated and untreated patients without symptoms (true plus false negatives). 95% Confidence intervals for sensitivity and specificity values were calculated using standard methods for determining confidence intervals of proportions [29]. Non-overlapping confidence intervals between two groups (or two time periods) indicate statistically significant differences in sensitivity and/or specificity.

Positive and negative likelihood ratios were calculated for both the intervention and comparison sites. These were calculated using mean values for sensitivity and specificity from the following formulas: Positive Likelihood Ratio (LR+) = sensitivity / (1-specificity); and Negative Likelihood Ratio (LR-) = (1-sensitivity) / specificity. LR+ constitutes the ratio of appropriately treated CAUTI (symptomatic) over the ratio of inappropriately treated ASB (asymptomatic). LR- constitutes the ratio of inappropriately non-treated CAUTI (symptomatic) over the ratio of appropriately not-treated ASB (asymptomatic). Using these definitions, we determined appropriate diagnosis of CAUTI using sensitivity, management of CAUTI (diagnosis and treatment) using LR+. Similarly, we determined appropriate diagnosis of ASB by specificity, and appropriate management of ASB using LR-.^28^

## Results and discussion

Table 1 demonstrates the baseline characteristics of study participants stratified by intervention and comparison sites. The Kicking-CAUTI study recruited 169 health care provider participants at the intervention site, and 65 similar providers at the comparison site. Most participants were resident physicians with fewer than three years of postgraduate training at both sites. There were no statistically significant differences between the providers at the two sites based on type of provider and level of training (Table 1).

**Table 1:** Participant characteristics by study site.

	Intervention Site N = 169	Comparison Site N = 65	P value
Type of provider ^a^			.08
Inpatient Providers	154 (91%)	62 (98%)	
Long-term Care Staff	15 (9%)	1 (2%)	
Level of training ^a^^,^ ^b^			
Resident physician, postgraduate year 1	76 (45%)	31 (49%)	.22
Resident physician, Postgraduate year 2	47 (28%)	18 (28%)	.98
Resident physician, Postgraduate year 3+	30 (18%)	13 (21%)	.83
Staff Physician	9 (5%)	1 (2%)	.29
Staff Nurse practitioner	3 (2%)	0	
Staff Physician Assistant	3 (2%)	0	

^a^Data missing from 2 participants at the comparison site;

^b^Data missing for 1 participant at the intervention site

Table 2 demonstrates the changes in diagnosis and treatment performance with cases of catheter associated bacteriuria confirmed as either CAUTI or ASB. Diagnosis and treatment accuracy were compared before and after algorithm implementation. At the intervention site, 129 cases were assessed in the pre-implementation period and 56 cases were assessed in the post-implementation period. At the comparison site, 67 cases were assessed in the pre-implementation period and 61 cases were assessed in the post-implementation period. Sensitivity was relatively high at both sites; however, there was a clinically important difference favoring the comparison site during pre-implementation (intervention site = 0.83, 95% confidence interval (CI): 0.73−0.94; comparison site = 0.90, 95%CI: 0.79−1.00) that remained post-implementation (intervention = 0.93, 95%CI: 0.83−1.00; comparison = 0.97, 95%CI: 0.92−1.00). Conversely, specificity was low at the intervention site prior to implementation (0.60, 95%CI: 0.50−0.71) and significantly improved in the post-implementation period (0.89, 95%CI: 0.78−1.00). Specificity was moderate (0.79, 95%CI: 0.66–0.92) at the comparison site pre-implementation and did not change (0.79, 95%CI: 0.63–0.95) post-implementation.

**Table 2:** Changes in provider accuracy with urinary tract infections (CAUTI) and Asymptomatic Bacteriuria (ASB) management (Diagnosis and treatment).

	Intervention Site				Comparison Site			
	Pre-Intervention		Post-Intervention		Pre-Intervention		Post-Intervention	
	Positive Cultures N = 129		Positive Cultures N = 56		Positive Cultures N = 67		Positive Cultures N = 61	
	Sxs	No Sxs	Sxs	No Sxs	Sxs	No Sxs	Sxs	No Sxs
Antimicrobials prescribed	40	32	26	3	26	8	36	5
Antimicrobials not prescribed	8	49	2	25	3	30	1	19
Sensitivity (95% CI)	83% (.73-.94)		93% (.83–1.00)		90% (.79–1.00)		97% (.92–1.00)	
Specificity (95% CI)	60% (.50-.71)		89% (.78–1.00)		79% (.66-.92)		79% (.63-.95)	
Positive* Likelihood Ratio	2.1		8.5		4.29		4.62	
Negative** Likelihood Ratio	0.28		0.08		0.13		0.04	

Sxs = Positive culture with symptoms; No Sxs = Positive culture without symptoms; N = number; CI = confidence interval

*Higher +LR raises the post-test probability and helps to (Rule-in) CAUTI diagnosis and encourage appropriate treatment of CAUTI.

**Lower–LR lowers the post-test probability and helps to (Rule-out) CAUTI diagnosis, and discourage treatment of ASB.

Positive and negative likelihood ratios, calculated from mean sensitivity and specificity rates, are described in Table 2. The LR+ at the comparison site did not change from pre- to post-implementation (4.29 to 4.62) but was higher pre-implementation compared with the intervention site. Similar to specificity, there was a clinically important improvement in LR+ at the intervention site from pre- to post-implementation (2.1 to 8.5). There were similar improvements in LR- at both sites from pre- to post-implementation, with the comparison site starting with a quantitatively lower LR- at pre-implementation compared to the intervention site (Table 2).

The results of this study demonstrate that diagnosis and treatment decisions related to catheter-associated bacteriuria can be substantially improved with adoption of a fast and frugal algorithm. Our findings suggest the algorithm improved participants’ accuracy of ASB diagnosis and CAUTI management as evidenced by significant improvements in specificity and LR+, respectively, at the intervention versus comparison site and when comparing post- to pre-implementation levels. As an important balancing measure for high specificity, very few false negatives cases (CAUTI cases that were not treated) were present after algorithm adoption at the intervention site. The improvement in CAUTI diagnosis and ASB management, measured by sensitivity and LR- rates, respectively, was similar at both the intervention and comparison sites when compared to pre-implementation levels. It is worth noting that inappropriate treatment of ASB markedly declined from 32 (40%) to 3 (11%) cases at the intervention site with adoption of the algorithm.

In additional to the EIT implementation model [21], the theoretical foundation for our Kicking-CAUTI algorithm [30] draws from two related bodies of literature (naturalistic decision making [31] and fast and frugal heuristics [32]) describing the intuitive judgement processes of experts. The overall approach of Kicking-CAUTI with its emphasis on aligning intuitive judgments of clinicians with CPGs through modeling with specific bacteriuria scenarios (e.g., audit-and-feedback using real, timely cases) is consistent with proscriptive evidence from naturalistic decision making [18]. The design of our algorithm itself is drawn specifically from the fast and frugal heuristics model [20, 32]. The search rule triggers the clinician to search through cues in a deliberate order whenever a catheter-associated bacteriuria case is encountered. Working down the algorithm, the first cue asks if there are any evidence-based symptoms of CAUTI. If the first cue is endorsed, then the second cue prompts the clinician to consider if there are any non-urinary causes that better explain these symptoms. The algorithm’s stop rule limits decision making to no more than these two cues to facilitate rapid judgments. The decision rule states that if the first cue is negative then ASB is the likely diagnosis. Conversely, if the first cue is positive and the second cue is negative, then the decision rule suggests that CAUTI is the likely diagnosis and a urine culture is warranted.

In the process of developing and piloting the algorithm [23,26], we identified that clinicians make consistent errors related to their diagnostic intuitions regarding catheter-associated bacteriuria. The design of the algorithm educates clinicians about common intuitive errors [23,33] and realigns diagnostic intuitions and treatment norms towards CPGs [31,32]. For example, in Fig 2, the algorithm provides a list of guidelines-concordant signs and symptoms diagnosing CAUTI. This list is placed right at cue 1 of the algorithm, which serves as a “fast and frugal” trigger for diagnostic intuitions. Furthermore, there is an explicit corrective about pyuria which we have previously shown to be a common yet incorrect rationale for ordering urine cultures [23,26]. These specific customizations may account for the improved specificity and LR+ in the current study and significant decline in overall rates of urine culture ordering in Kicking CAUTI [25].

The current study has limitations. The study was conducted at two United States Veterans Health Care Systems (VA) in Texas. Therefore, our results may not generalize beyond this region or to private healthcare systems. However, the VA is cited frequently for leading quality improvement innovations to reduce catheter related infections. Further, the comparison site activities in the current study included their strong prior and ongoing quality improvement efforts, which may explain the pre-implementation differences at this site. In many cases, the case based training was offered to teams of clinicians rather than individuals. However, identification of cases, training protocols, and use of the algorithm to guide decisions were all based on standardized procedures to ensure greater reproducibility of our methods and results. In addition, there is potential for bias arising from the non-randomized, quasi-experimental design. Effective matching of participant characteristics at both sites and the use of matched baseline and intervention periods may address some sources of bias, but not all as noted by higher baseline sensitivity and specificity at the comparison site. The process of case classification at both sites to define the criterion standard for CAUTI and ASB may have some error, but should not have introduced bias. However, we validated and standardized our case classification process with good inter-rater agreement between sites for the overall Kicking CAUTI study [25,27].

## Conclusions

Overtreatment of ASB is common and reflects poor adherence with clinical guidelines for the management of catheter associated bacteriuria. Prior attempts at translating guidelines into routine care have relied on complex, multicomponent interventions that are difficult to sustain and disseminate. Using the Evidence Integration Triangle (EIT), we have demonstrated sustained reductions in urine culture ordering and treatment of ASB following implementation of Kicking CAUTI at the intervention site [25]. The current study reports on improvements in diagnosis and treatment decision making for individual bacteriuria cases among clinicians at the intervention versus comparison site. This was achieved through the use of an algorithm based on fast and frugal heuristics within a naturalistic (rather than economic) approach to decision-making [32–33]. Our study findings suggest that understanding and shaping clinician behavior through the paradigm of experts’ intuitive judgments may be a more effective approach for rapidly translating evidence into routine care.
